# Is Postoperative Imaging Mandatory after Meningioma Removal? Results of a Prospective Study

**DOI:** 10.1371/journal.pone.0124534

**Published:** 2015-04-27

**Authors:** Florian Geßler, Stephan Dützmann, Johanna Quick, Karima Tizi, Melanie Alexandra Voigt, Haitham Mutlak, Hartmut Vatter, Volker Seifert, Christian Senft

**Affiliations:** 1 Department of Neurosurgery, University Hospital Frankfurt, Goethe-University, Schleusenweg 2–16, 60528, Frankfurt, Germany; 2 Institute of Neuroradiology, University Hospital Frankfurt, Goethe-University, Schleusenweg 2–16, 60528, Frankfurt, Germany; West German Cancer Center, GERMANY

## Abstract

**Background:**

Routine postoperative imaging (PI) following surgery for intracranial meningiomas is common practice in most neurosurgical departments. The purpose of this study was to determine the role of routine PI and its impact on clinical decision making after resection of meningioma.

**Methods:**

Patient and tumor characteristics, details of radiographic scans, symptoms and alteration of treatment courses were prospectively collected for patients undergoing removal of a supratentorial meningioma of the convexity, falx, tentorium, or lateral sphenoid wing at the authors’ institution between January 1st, 2010 and March 31st, 2012. Patients with infratentorial manifestations or meningiomas of the skull base known to be surgically difficult (e.g. olfactory groove, petroclival, medial sphenoid wing) were not included. Maximum tumor diameter was divided into groups of < 3cm (small), 3 to 6 cm (medium), and > 6 cm (large).

**Results:**

206 patients with meningiomas were operated between January 2010 and March 2012. Of these, 113 patients met the inclusion criteria and were analyzed in this study. 83 patients (73.5%) did not present new neurological deficits, whereas 30 patients (26.5%) became clinically symptomatic. Symptomatic patients had a change in treatment after PI in 21 cases (70%), while PI was without consequence in 9 patients (30%). PI did not result in a change of treatment in all asymptomatic patients (p<0.001) irrespective of tumor size (p<0.001) or localization (p<0.001).

**Conclusions:**

PI is mandatory for clinically symptomatic patients but it is safe to waive it in clinically asymptomatic patients, even if the meningioma was large in size.

## Introduction

Intracranial meningiomas account for approximately 20–25% of all intracranial tumors[1], and surgical removal usually is the preferred method of treatment. Although the value of pre- and intraoperative imaging in meningioma surgery has been described extensively[2–7], the impact of routine postoperative computed tomography (CT) or magnetic resonance imaging (MRI) remains controversial, especially in patients without any postoperative deficit. Literature on postoperative meningiomas is scarce[8] and limited to certain areas[9] and populations[10]. Because neurological examination may be impaired in patients after craniotomy due to anesthetics, analgesics or swelling, routine early postoperative imaging (PI) is often ordered. The rationale behind this neuroimaging following meninigoma surgery is to identify emerging problems such as bleeding or infarction at an early stage. In most cases the impact on clinical decision-making may be limited.

The goal of this study was to determine the necessity of these postoperative scans in patients suffering from surgically easily accessible intracranial meninigomas. Whereas PI is often necessary for the planning of postoperative treatment and follow-up studies of partially resected meningiomas and meningiomas of difficult localization, we hypothesize that PI following uneventful resection in patients with meningiomas in easily accessible location is of limited value, and thus can be safely waived.

## Materials and Methods

We included adult patients of both sexes who underwent surgery via a craniotomy with easily accessible meningioma localization between January 1, 2010 and March 31, 2012. Meningiomas of the following localizations were defined as easily accessible: meningioma of the convexity, the cerebral falx, the supratentorial part of the tentorium or the lateral part of the sphenoid wing. Patient data consisting of gender, age, localization and size of meningiomas, modality and findings of PI, postoperative complications and their treatment were prospectively collected and entered into a database (patient details S1 Table).

Postoperatively, patients were admitted to the neurosurgical ICU and observed until postoperative day one. Routine PI was performed on postoperative day one, symptomatic patients underwent imaging after onset of symptoms. The modality of PI was chosen according to the surgeon’s preference.

We reviewed PI findings for intracranial air, subdural effusion, epidural hematoma or perilesional edema. The consequences of PI with respect to changes in therapeutic regimen were recorded as well. Routine postoperative procedures included neurosurgical ICU monitoring for 12–24 hours, application of steroids for 5 days and application of osmodiuretics for 3 days. Changes in postoperative treatment were defined as intensified antiedematous treatment, return to the intensive care unit or return to the operating room. For patients who received CT instead of MRI we calculated the region specific normalized radiation exposure to assess postoperative exposure to iatrogenic radiation.

Statistical analyses were performed using commercially available software (SPSS Statistics 12, IBM, Chicago, IL). Differences between dichotomized variables were calculated using Fisher’s exact test. Differences between CT and MR imaging in regard to predicting the need for a change in the postoperative routine were calculated using McNemar’s test. P values lower than 0.05 were considered statistically significant.

The Goethe University Clinics ethics committee Frankfurt, Germany approved conduction of this study (approval no. 4/09, project SNO_NCH_02–10), and all patients gave written informed consent into the collection of data.

## Results

Between January 2010 and March 2012, we performed surgery on 206 patients with intracranial meningiomas. WHO grades I-III using microsurgical techniques. Of all patients, 2 patients did not receive postoperative imaging and were excluded from the study. 91 patients had a meningioma in difficult location and 113 in easily accessible location, who were included in this study.

Of these patients, median age at surgery was 59 years (range: 31–84 years), 38 patients were of male and 75 patients of female gender (m:f ratio 1:1.97)).

**Fig 1:**
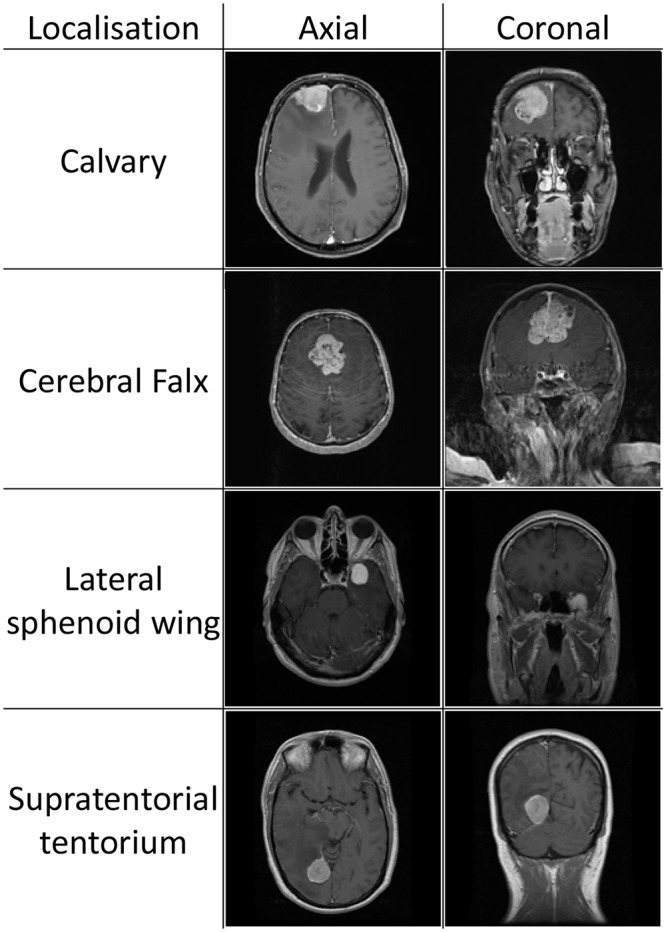
Meningioma location. Exemplary images of meningioma location to demonstrate assessment of location of the tumor as easily accessible in this study.

We analyzed 51 patients with meningiomas of the convexity (45%), 29 patients with meningiomas of the cerebral falx (26%), 10 patients with meningiomas of the supratentorial part of the tentorium (9%) and 23 patients with meningiomas of the lateral sphenoid wing (20%) (Fig 1). PI consisted of CT in 73 patients and of MRI in 40 patients.

The maximum tumor diameter measured < 3 cm in 25 patients (22%), 3–6 cm in 70 patients (62%) and > 6 cm in 18 patients (16%).

**Fig 2:**
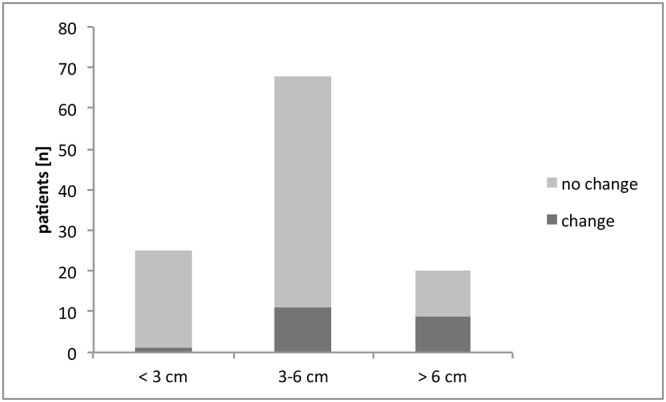
Distribution of change of treatment on ICU patients dependent on tumor size. Patients were assigned to the indicated groups dependent on meningioma size. Tumor size of > 6 cm is significantly more often associated with a change in postoperative treatment compared to a tumor size of < 3 cm (p<0.005) and 3–6 cm (p<0.05, Fisher’s exact test)

**Fig 3:**
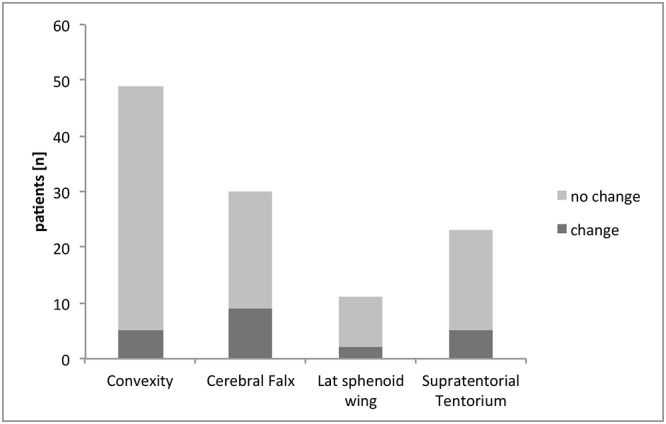
Distribution of change of treatment on ICU patients dependent on tumor localization. Patients were assigned to the indicated groups dependent on meningioma location. The occurrence of a change in ICU treatment did not differ between tumor location (Fisher’s exact test).

83 patients were in unremarkable postoperative status without displaying any new neurological symptoms, whereas 30 patients had new clinical symptoms (Table 1). The initial symptoms that were observed in symptomatic patients were prolonged waking up from anesthesia in 5 patients (17%), seizure in 5 patients (17%), new or worsened pre-existing neurological deficit in 18 patients (60%) or other in 2 patients (6%). As displayed (Fig 2), surgery of a large meningioma was associated with a significantly higher rate of patients in need for change in postoperative ICU regimen whereas tumor localization did not display to be of an influence on the need for change in the ICU regimen (Fig 3)[4].

**Table 1:** Distribution of initial symptoms of symptomatic patients.

Symptomatic patients	n = 30	
prolonged awakening	4	13%
seizure	5	17%
neurological deficit	18	60%
other	3	10%

Distribution of all 30 symptomatic patients, displaying initial pathological symptoms as prolonged awakening, seizure, and a new neurological deficit. The patients summarized as “other” displayed personality change in two cases and gait disturbance in another case.

Of the 83 patients with unremarkable postoperative status, 56 had postoperative CT scans whereas 27 underwent MR imaging. In these patients, PI displayed intracranial air in 51 patients (61%), small subdural effusion in 67 patients (81%), small epidural hematoma in 6 patients (7%) and perilesional edema in 77 patients (93%). None of the radiologic findings caused a mass effect. Overall, in none of the 83 clinically impaired patients a change in postoperative treatment as defined before was done.

Postoperative imaging of the 30 newly symptomatic patients consisted of CT in 18 patients and of MRI in 12 patients. The imaging displayed intracranial air in 28 patients (93%), subdural effusion in 27 patients (90%), epidural hematoma in 1 patient (3%) and perilesional edema in 29 patients (97%). A mass effect of subdural hematoma was observed in 2 patients (7%) (Table 2). For this study, all patients with radiographic findings of subdural air were included. All cases of pneumocephalus were cases of simple pneumocephalus without signs of tension pneumocephalus (i.e. Mt. Fuji sign or air bubble sign) and therefore no invasive or non-invasive measures as high oxygen ventilation were performed in all of the cases observed.

**Table 2:** Radiographic findings in postoperative imaging after meningioma surgery.

Radiographic finding	Asymptomatic patients (n = 83) [n]	Symptomatic patients (n = 30) [n]
Pneumocephalus	51 (61%)	28 (93%)
Subdural hematoma	67 (81%)	27 (90%)
Epidural hematoma	6 (7%)	1 (3%)
Perilesional edema	77 (93%)	29 (97%)

Radiographic findings in both clinically symptomatic and asymptomatic patients

As a consequence to the imaging in symptomatic patients, 14 patients (46.7%) were monitored > 24 hours on the neurosurgical ICU, 18 patients (60%) received intensified antiedematous medication, 2 patients (7%) were brought to the operating theater for repeat surgery. No change in clinical treatment was necessary in 7 patients (23%). Both patients receiving repeat surgery did display hematoma with mass effect. PI led to an alteration of postsurgical treatment only in symptomatic patients whereas no change in postoperative treatment was necessary in the group of clinically unimpaired patients (Table 3).

**Table 3:** Therapy in symptomatic patients.

Symptomatic patients	n = 30	
no consequence	9	30%
prolonged ICU stay	14	46.7%
antiedematous treatment	9	30%
surgery	2	7%

Post-imaging therapy of all 30 patients who had new signs or symptoms postoperatively.

Symptoms in the patients were detected during the postoperative treatment on the neurosurgical ICU (<24 h postoperatively). All patients were first symptomatic, and than a postoperative scan was performed. None of the patients with pathological imaging without clinical symptoms was in the need for treatment for his or her pathological finding; treatment was necessary only when patients were symptomatic in any way.

Both patients undergoing repeat surgery displayed newly diagnosed postoperative neurological deficits as their initial symptom. Postoperative imaging was obtained in postoperative hours 3 and 8. In both cases subdural hematomas developing mass effects were displayed in postoperative imaging obtained. One of the two patients had a regular postoperative course, being able to be discharged from ICU on p.o. day one; the other patient had a prolonged stay on the ICU for 8 consecutive days. At follow-up after 3 months, none of the patients requiring repeat surgery had a persistent neurological deficit.

In summary, 113 patients underwent postoperative imaging after resection of a meningioma commonly regarded as surgically easily accessible. In only 3 patients (3%), both the postoperative scan displayed unremarkable results and no change of treatment was necessary. No postoperative imaging displayed a false negative result defined as imaging without pathological results but a change in the postoperative treatment regimen. The opposite trend was far more prevalent with 110 patients (97%) who underwent postoperative imaging revealing a pathological finding. Of these patients, only 23 patients required a change in the routine of postoperative treatment, whereas only 6 patients displayed a permanent neurological deficit on follow-up after 3 months (Table 4).

**Table 4:** Characteristics of patients suffering deficits at follow-up.

Patient	Tumor size	Tumor location	Deficit	Onset	Etiology
**1**	> 6 cm	Cerebral falx	Paresis	postop.	ischemic infarction
**2**	3–6 cm	Cerebral falx	Paresis	postop.	ischemic infarction
**3**	3–6 cm	Convexity	Paresis	postop.	Injury during removal of infiltrating tumor
**4**	3–6 cm	Convexity	Speech disorder	postop.	ischemic infarction
**5**	3–6 cm	Convexity	Paresis	postop.	Injury during removal of infiltrating tumor
**6**	< 3 cm	Convexity	Paresis	preop.	Injury during removal of infiltrating tumor

Characteristics of patients suffering from permanent neurological deficits. Patients were reevaluated after follow up of 3 months.

We calculated sensitivity and specificity of PI for predicting the need for a change of the routine postoperative treatment was calculated. CT had a sensitivity of 100% (CI 75.8%- 100%) and a specificity of 2% (CI 0,3%- 9%). MRI had a sensitivity of 100% (CI 70.11%- 100%) and specificity of 6,5% (CI 1.8%- 20.7%). When comparing CT and MRI, there was no difference in predicting the need for extraordinary postoperative treatment in symptomatic patients (p < 0.01).

Radiation exposure of all patients who underwent cranial computed tomography was calculated using the E_DLP_ (region specific normalized effective dose, 0,0023 mSv mGy^-1^ cm^-1^). Analysis of all patients who underwent computed tomography displayed a median of 725 mGy per scan (range: 662–757 mGy).

## Discussion

In other surgical specialties such as urology and maxillofacial surgery, there is research in progress displaying the limited value of postoperative imaging [11, 12]. Also, neurosurgeons have questioned standard pre- and postoperative procedures[13] and demonstrated limited value of routine PI in craniosynostosis surgery[14] and shunt series[15]. Although it is common practice to obtain postoperative imaging in patients receiving surgery on intracranial lesions, the displayed findings often do not have clinical significance. In glioma surgery, contrastingly, early PI by means of MRI is mandatory to determine the extent of resection and to serve as a basis for later follow-up studies[16]. The extent of resection achieved in meningioma surgery, however, can be reliably established by the surgeon himself[17]. Whereas the role of imaging in the long-term follow up of meningioma patients is well established[18] in regards to follow-up, the necessity of early PI remains unclear. In this series, we have collected patients for which the administration of PI might be discussed in clinical practice every day.

Without doubt, cranial imaging has an impact on patients with meningioma involving eloquent brain areas and of complex formation. Early imaging of partially resected meningiomas, expecially if not WHO grade I, is of importance for further postoperative therapy. In the ICU setting, CT and MR imaging generally are of high value, helping in clinical decision-making. Due to the broad availability of CT imaging in particular and due to the surgeons’ need not to miss postoperative intracranial pathologies, PI has become an unquestioned routine after meningioma surgery in most neurosurgical departments.

For a cranial CT the mean effective dose after radiation exposure was around 1,6 mSv. Minimizing exposure to radiation is mandatory because radiation exposure leads to an increased lifetime cancer mortality risk[19] as it has been demonstrated in regard to head CT[20]. This accounts especially for patients with intracranial meningiomas, since these patients harbor benign tumors and receive multiple cerebral imaging from diagnosis to follow up[21]. Moreover, postoperative CT is insufficient for the follow-up of meningioma patients[18]. Thus, routinely ordered postoperative MRI is desirable, but not feasible for practical and financial reasons, with the MRI duration being longer, its availability relatively limited, and its costs being usually more than twice the cost of a CT. Limited resources mandate meaningful allocation of diagnostic imaging. Although CT allows fast and relatively cheap imaging, MRI, on the other hand, provides wider diagnostic spectrum including postoperative ischemic changes that cannot be seen in early CT. Although routinely performed in most neurosurgical centers, the length of the study and high magnetic field create technical issues. Every scan should therefore be justified and chosen on an individual basis.

Of note, two patients underwent repeat surgery for postoperative hemorrhage. Both patients were clinically symptomatic and recovered well. Still it remains to be discussed, if “very early” postoperative imaging (directly postoperative/after arrival on ICU) may have identified both patients before developing clinical symptoms. In the end, the timing of postoperative hemorrhage after meningioma surgery remains speculative and “early” postoperative imaging may either display no hemorrhage at all or miss a developing mass effect. In both cases, repeat imaging would have been necessary as a patient becomes symptomatic.

We found that PI was of little impact in patients with easily accessible meningiomas and unremarkable postoperative clinical courses. In our series, 83 clinically asymptomatic patients received either postoperative CT or MR imaging, which did not display clinically relevant findings or led to any change in routine postoperative treatment. The indication for early postoperative imaging may therefore be questioned as these patients might receive radiation dose, if CT is chosen, and are a burden to departmental resources. Criticial questioning may arise in regard to the question whether imaging in an initially asymptomatic patient could allow a more timely initiation of the appropriate management, even prior to an onset of symptoms. We have not identified a single patient in more than two years and over 150 procedures who would have demonstrated a benefit by this procedure under the named setting of postoperative ICU care.

In contrast, PI displayed pathological findings in the vast majority of patients with new signs or symptoms following meningioma resection, and PI led to therapeutic consequences in three out of four affected patients, thus highlighting the necessity of PI in symptomatic patients.

Overall, 30 patients displayed new postoperative symptoms, which is line with published data[22]. Of these 30 patients a majority of 24 patients recovered of their initial symptoms resulting in 6 patients with a permanent neurological deficit after follow up. These data concur with the literature and underline that surgery on meningioma is not without remaining morbidity although easily accessible[23].

As a consequence of this study, a policy change concerning the postoperative routines has taken place at the authors’ institution: we have ceased routine early PI in asymptomatic patients following surgery of easily accessible meningiomas, but keep PI reserved for symptomatic patients only.

For legal issues one might still argue in favor of routine PI for all patients, as after further observation of an even larger group of patients one may experience a clinically asymptomatic patient who does not receive PI but becomes symptomatic in the later postoperative course. Therefore, we have been continuing data collection in asymptomatic patients without PI in order be able to provide results on the long-term safety of this treatment concept in the future.

This study further shows that CT and MR imaging are of equal value in terms of prompting changes to the postoperative care in clinically symptomatic patients, so the imaging modality can be chosen on an individual basis taking into account institutional, logistical, as well as patient specific aspects.

Our study has several limitations: One of the major aspects is the possible case-selection bias. Because we only included patients with easily accessible meningiomas, a substantial number of patients undergoing meningioma surgery was excluded. On the other side, all patients were operated in the same institution and inclusion criteria for this study were well defined, as the selection reflects the clinical routine. Secondly, the study is underpowered in regard to adequate differences between CT and MRI imaging despite the known benefits of MR imaging as better resolution to detect small lesions or ischemia at an early stage.

## Conclusions

The need for routinely performed early PI in asymptomatic patients undergoing meningioma removal is questionable. Patients suffering from pathological postoperative events must receive CT or MRI for therapeutic and forensic reasons. It may be justified to refrain from PI in asymptomatic patients after removal of intracranial meningioma in easily accessible localization.

## Supporting Information

S1 Table Raw de-identified data for each patient analyzed for this manuscript. Characteristics of all patients included for analysis.
(XLS)
